# Dr. Abraham M. Owen

**Published:** 1898-10

**Authors:** 


### OBITUARY.

Dr. Abraham M. Owen, of Evansville, Ind., died suddenly in that city, September 18, 1898, aged 49 years. He had not been in his usual health for about a year, yet neither himself nor his friends supposed he was in an immediately critical condition. He retired in good season and in good spirits on the night of the 17th, but was discovered breathing heavily and in apparent distress shortly before 3 o'clock a. m. His associate, Dr. Edwin Walker, was summoned, but before he could reach the bedside Dr. Owen became unconscious and death came very soon.

Dr. Owen was one of the best known physicians in the United States. His father was a physician and the son early exhibited a desire to pursue the same profession. After spending some of his student life in his father's office, he went to New York and further pursued his studies as a pupil of Professor Frank Hastings Hamilton, M. D. He graduated at Bellevue Hospital Medical College in 1870, and immediately thereafter located at Evansville, where he continued the practice of his profession until his death. He attained unusual prominence as a physician early in his career and later he became famous as a skilful surgeon. He took an active part in the local, state and national medical societies and was named as treasurer-general of the first Pan-American Medical Congress that met at Washington in 1893.

Dr. Owen was gifted in many ways. In additon to his professional accomplishments he was an easy debater, a graceful speaker—witty, logical and convincing; a delightful companion at dinner or in the drawing-room; a useful and energetic citizen, a most lovable man. A widow and three children—a daughter and two sons—survive; also a sister and two brothers, all of whom receive the sympathy of many hundreds of people throughout the country.

